# Metabolomic Profiling of Plasma in Children with Drug-Resistant Epilepsy: The Effect of the Ketogenic Diet on Energy and Amino Acid Pathways

**DOI:** 10.3390/nu18142347

**Published:** 2026-07-17

**Authors:** Marta Cieślak, Paulina Gątarek, Piotr Safiński, Lukasz Przyslo, Joanna Kałużna-Czaplińska

**Affiliations:** 1 Institute of General and Environmental Chemistry, Faculty of Chemistry, Lodz University of Technology, 116 Zeromskiego Street, 90-924 Lodz, Poland; 248059@edu.p.lodz.pl; 2 Department of Developmental Neurology and Epileptology, Research Institute of Polish Mother’s Memorial Hospital, 93-338 Lodz, Poland; piotr.safinski@iczmp.edu.pl (P.S.); lukasz.przyslo@iczmp.edu.pl (L.P.)

**Keywords:** ketogenic diet, drug-resistant epilepsy, gas chromatography–mass spectrometry, plasma metabolome

## Abstract

Background: Drug-resistant epilepsy in children remains a major therapeutic challenge, and the limited efficacy of pharmacological treatment highlights the need for alternative therapeutic approaches, including the ketogenic diet (KD). Although the clinical efficacy of KD is well established, its metabolic effects are not fully understood. This study aimed to characterize the plasma metabolomic profile of children with drug-resistant epilepsy and to explore metabolic differences associated with ketogenic dietary therapy. Methods: Eighteen children with drug-resistant epilepsy were enrolled, including nine receiving KD and nine receiving a standard diet. Plasma metabolomic profiling was performed using gas chromatography-mass spectrometry (GC-MS). Data were analyzed using multivariate (PCA and OPLS-DA) and univariate statistical approaches. Both variable importance in projection (VIP) scores and metabolic pathway analysis were used to support biological interpretation. Results: A total of 462 annotated GC-MS features were retained after data filtering. OPLS-DA demonstrated clear separation between study groups (R^2^Y = 0.937; Q^2^ = 0.594). Eighty-three metabolites had VIP scores > 1, indicating a substantial contribution to the OPLS-DA model, while 22 metabolites differed between groups based on unadjusted *p*-values. However, only three metabolites remained statistically significant after false discovery rate correction. After false discovery rate correction, β-hydroxybutyrate and citric acid remained significantly higher in the KD group, whereas 1,4-butanediol was significantly lower. Lower glucose and tryptophan levels were observed only in the unadjusted analyses and should therefore be regarded as exploratory. Similarly, the pathway analysis findings, including those related to amino acid metabolism, were exploratory and require confirmation in larger cohorts. Conclusions: Ketogenic dietary therapy was associated with distinct changes in the plasma metabolomic profile of children with drug-resistant epilepsy. While the most robust findings involved β-hydroxybutyrate, citric acid, and 1,4-butanediol, the observed changes in glucose and tryptophan, as well as the amino acid-related pathway findings, should be considered exploratory and require validation in larger independent cohorts before being interpreted as biomarkers or evidence of specific metabolic mechanisms.

## 1. Introduction

Epilepsy is considered the most common chronic and paroxysmal neurological disorder of childhood, characterized by unprovoked, recurrent convulsive and non-convulsive seizures caused by excessive neuronal activity and excitation in the central nervous system [1]. It is estimated that at least 60 out of every 100,000 people are children under the age of 5. Furthermore, in the first 3 years of life, 35% of children develop drug-resistant epilepsy [2]. Drug-resistant epilepsy (DRE) affects nearly 30% of patients and poses a significant clinical challenge in selecting appropriate and effective treatment [3]. Epilepsy begins during childhood, and particularly its drug-resistant form, raises many concerns and doubts regarding the health of young patients. Intense and recurrent electrical discharges in nerve cells have a direct impact on the functioning of children and their families. Cognitive impairment and an increased risk of death are among the most serious effects of long-term epileptic seizures [4]. In addition to the health effects, young patients also experience psychosocial difficulties. Recurrent and unexpected seizures cause limitations in daily functioning through social stigmatisation, complications in peer relationships and disruption to normal school attendance. Difficulties with learning and high levels of school absenteeism can lead to a deterioration in patients’ quality of life and self-esteem and hinder the proper development of interpersonal relationships [5].

Selecting effective and safe treatment poses a challenge for paediatric neurologists, neurologists and epileptologists. Available traditional forms of therapy include pharmacological methods (mono- and polypharmacotherapy) and non-pharmacological methods, which include epilepsy surgery, the use of neurostimulation techniques (VNS, DBS) and the implementation of a ketogenic diet [4,6,7]. These approaches have been reported to improve quality of life in 40–80% of patients, depending on the form of therapy [4,7]. The limited effectiveness of pharmacological treatment increasingly necessitates the use of non-pharmacological approaches, including the ketogenic diet (KD) as a drastic dietary intervention that is thought to influence mechanisms involved in seizure generation [8,9]. Despite its documented clinical efficacy, the ketogenic diet remains the subject of ongoing research to investigate more precisely its effect on inhibiting epileptogenesis and epileptic activity. Among the proposed and analysed mechanisms are, amongst others, metabolic changes in the mitochondria of pre- and postsynaptic neurons and in the environment of astrocytes and microglia, where the production of ketone bodies reduces oxidative stress and modulates amino acid metabolism, thereby enhancing inhibitory GABAergic transmission [10,11,12].

In the ketogenic diet, a drastic reduction in sugar intake induces characteristic changes in energy metabolism, including the Krebs cycle. β–oxidation of fatty acids occurring in the mitochondria of liver cells leads to increased synthesis of ketone bodies such as β-hydroxybutyrate (BHB), acetoacetate and acetone [8,13,14,15,16]. BHB, a key marker of ketosis during ketogenic dietary therapy, serves as an alternative energy source and has also been proposed to influence neuronal membrane stabilization and the expression of genes involved in neurotransmitter metabolism (e.g., GAD1 and SIRT4) [16,17].

**Figure 1:**
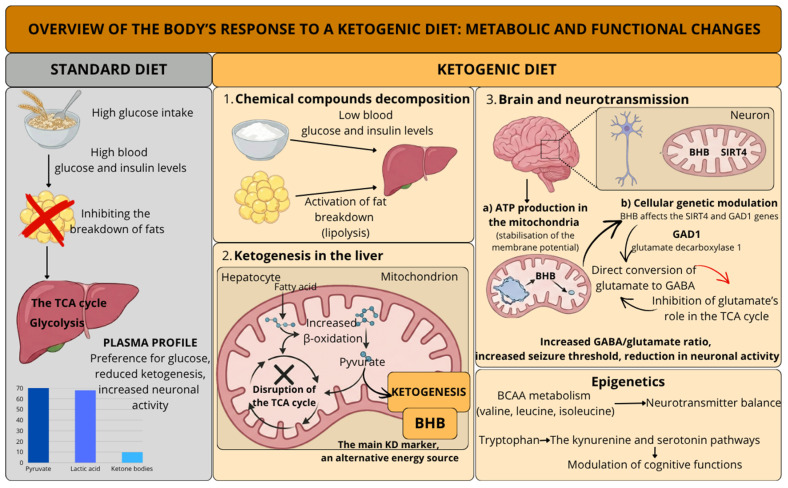
An overview of the body’s response to a ketogenic diet: metabolic and functional changes.

In parallel with modifications to the energy pathway, the influence of the ketogenic diet is also evident in the metabolism of branched-chain amino acids (BCAAs). This group comprises three amino acids—valine, leucine and isoleucine—which are responsible for regulating neuronal activity and the transport of tryptophan to nerve cells [18,19]. Changes in BCAA metabolism indirectly affect both the kynurenine pathway and the serotonin pathway, whilst also influencing nervous system activity via epigenetic factors [11,20]. An overview of the principal metabolic and functional changes associated with the ketogenic diet is presented in Figure 1.

In recent years, metabolomics has become a key research tool in the study of the pathophysiology of neurological diseases. This field enables a comprehensive analysis of low-molecular-weight compounds present in patients’ body fluids and tissues. The metabolome is a collection that is extremely sensitive to changes in the body’s internal environment. This means it reflects the patient’s actual condition, and its documented potential has become a hallmark of research into diseases, including drug-resistant epilepsy. One of the commonly used analytical techniques in metabolomic studies is gas chromatography coupled with mass spectrometry (GC-MS). Simultaneous identification and quantitative analysis enable the examination of a broad spectrum of metabolites involved in biochemical reactions.

Previous metabolomic studies suggest that a ketogenic diet may lead to significant changes in plasma and urine metabolic profiles [10,11]. These changes include, amongst others, the metabolism of fatty acids, amino acids and pathways related to energy production [10,11,12]. However, the limited number of published studies on drug-resistant epilepsy and the role of the ketogenic diet in the therapeutic process makes it difficult to verify the hypotheses put forward. Currently, there is little data showing differences in metabolic profiles between patients on a ketogenic diet and those on a high-carbohydrate diet. At the same time, there is a lack of information linking the metabolic profile to the type and frequency of epileptic seizures.

The aim of this study was to characterize the plasma metabolic profiles of children suffering from drug-resistant epilepsy and to assess the impact of the ketogenic diet on selected aspects of metabolism, including energy and amino acid metabolism. Attention was paid to identifying metabolites that distinguish patients on a ketogenic diet from those on a standard diet, using gas chromatography coupled with mass spectrometry.

## 2. Materials and Methods

### 2.1. Chemicals and Reagents

N,O-Bis(trimethylsilyl)trifluoroacetamide (BSTFA), methoxyamine hydrochloride, and pyridine were purchased from Sigma-Aldrich Inc. (St. Louis, MO, USA). LC- or GC-grade water, isopropanol, and acetonitrile were purchased from Merck (Darmstadt, Germany).

### 2.2. Clinical Samples

**Figure 2:**
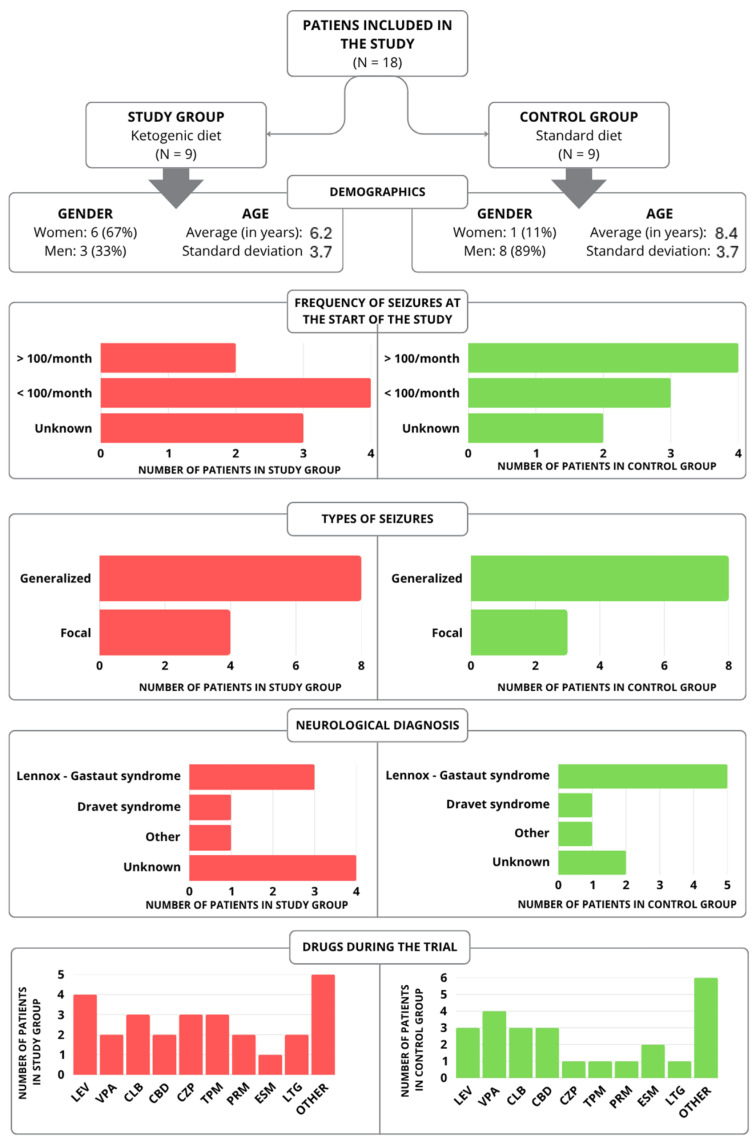
Patient characteristics, including diagnosis and medications taken. LEV—levetiracetam; VPA—valproic acid; CLB—clobazam; CBD—cannabidiol; CZP—clonazepam; TPM—topiramate; PRM—primidone; ESM—ethosuximide; LTG—lamotrigine.

The study included 18 patients diagnosed with drug-resistant epilepsy from the Research Institute of Polish Mother’s Memorial Hospital in Łódź. The study group consisted of 9 children on a ketogenic diet (either a modified Atkins diet or a classic ketogenic diet), whilst the control group comprised patients on a standard diet. The characteristics of the groups are presented in Figure 2. The study was conducted in accordance with legal requirements. The Bioethics Committee at the Polish Mother’s Memorial Hospital Research Institute granted approval for the study (Resolution of the Bioethics Committee at the ‘Polish Mother’s Health Centre’ Institute No. 1/2026 of 27 January 2026).

### 2.3. Ketogenic Dietary Treatment

Patients received dietary therapy according to clinical indications and individual nutritional requirements. Depending on the therapeutic protocol, participants followed either a classical ketogenic diet (KD), modified Atkins diet (MAD), or low glycemic index treatment (LGIT). The duration of dietary therapy before blood collection ranged from 2 to 258 weeks. Detailed meal plans were reviewed by a dietitian and modified when necessary to maintain compliance with the prescribed dietary protocol. In patients receiving classical KD, ketogenic ratios ranged from 1.5:1 to 3:1. Daily energy intake was individualized according to age, body weight, nutritional status, and clinical requirements and ranged from approximately 760 to 1660 kcal/day. Protein intake targets were also individualized and ranged from approximately 13.5 to 42 g/day.

All dietary prescriptions and meal plans were supervised by an experienced dietitian and adjusted when necessary. Dietary adherence was monitored using dietary records (Ketoplaner), regular dietary consultations, and routine measurements of blood β-hydroxybutyrate (BHB) concentrations. In selected cases, urinary ketones were also assessed as part of routine clinical monitoring. Blood samples for metabolomic analyses were collected after an overnight fast.

Detailed quantitative dietary intake records, including complete macronutrient distributions and specific protein sources, were not available for all participants. Therefore, although dietary therapy was actively supervised and ketosis was routinely monitored, the potential contribution of individual dietary components, including protein composition, to the observed metabolomic differences cannot be completely excluded.

### 2.4. Sample Preparation

The plasma samples were stored at −28 °C. The sample preparation method was based on the method proposed by O. Fiehn [21]. Frozen plasma was thawed on ice. The samples were then mixed and centrifuged at 13,000 RCF for 2 min. From the plasma prepared in this way, 90 µL was taken and transferred to a 2 mL Eppendorf tube. The next stage involved deproteinising the samples using a previously prepared ACN/IPA/H_2_O solution (3:3:2, *v*/*v*/*v*). 1 mL of the cold mixture was added to the plasma, mixed, and placed on a shaker for 5 min. After this time, the samples were centrifuged at 13,000 RCF for 2 min. 450 µL of the supernatant was transferred to a chromatography vial and dried under a nitrogen atmosphere at 37 °C. The dry residue was subjected to a two-step derivatisation. In the first step, 10 µL of methoxyamine hydrochloride in pyridine (20 mg/mL) was added and the mixture was incubated on a heating block for 30 min at 37 °C. In the second stage, 90 µL of BSTFA was added and the mixture was incubated again for 30 min at 37 °C. The prepared samples were transferred to GC vials with an insert and subjected to GC-MS analysis. All study samples, analytical replicates, pooled biological quality-control (QC) samples, reagent blanks, and method blanks were prepared, derivatized, and analyzed as a single analytical batch. Pooled QC samples were used to monitor analytical reproducibility and instrument stability, whereas blank samples were included to identify potential background and contamination-related signals.

### 2.5. Gas Chromatography–Mass Spectrometry Analysis

To determine metabolites in the plasma, samples were analysed by GC–MS. For the derivatized samples, 1 μL of aliquot was injected in the splitless mode using an autosampler into an Agilent 7890A gas chromatograph (Agilent Technologies, Santa Clara, CA, USA) equipped with HP-5MS column (30 m length, 0.25 mm ID and 0.25 μm film thickness). The injector temperature was set to 280 °C. The constant flow of 1 mL/min was set through the column, and helium was used as a carrier gas. The column temperature was initially kept at 70 °C for 1 min and then increased from 70 °C to 300 °C at 12 °C/min, where it was held for 14 min. The transfer line temperature was set to 280 °C and the ion source temperature at 230 °C. Ions were generated by standard electron ionization energy of 70 eV. Masses were acquired from *m*/*z* 50 to 635. The total run time was 34 min and 10 s.

### 2.6. Statistical Analysis

Metabolomic data analysis was performed using MetaboAnalyst 6.0 (https://www.metaboanalyst.ca/, accessed on 15 June 2026). The input data were normalised to the sum, subjected to cube root transformation, and scaled using the Pareto scale. First, a PCA analysis was performed to visualise the overall distribution of patient profiles in the study and control groups. To examine group discrimination in greater detail, Orthogonal Partial Least Squares Discriminant Analysis (OPLS-DA) was performed. Model performance was evaluated using the explained variance in the predictor matrix (R^2^X), the explained variance in the response variable (R^2^Y), and predictive ability (Q^2^). To assess model robustness and reduce the risk of overfitting, permutation testing with 1000 permutations was performed.

In addition, recursive support vector machine (SVM) classification was applied as an independent validation approach using 10-fold cross-validation (CV), leave-one-out cross-validation (LOOCV), and bootstrap validation. Classification error rates obtained from these validation strategies were used to assess model stability and predictive performance. Variable importance in projection (VIP) scores derived from the OPLS-DA model were used to identify metabolites contributing most strongly to group discrimination.

Metabolites with a VIP score above 1 were considered important contributors to group discrimination in the OPLS-DA model, and the resulting panel of compounds was compared with the results of Student’s *t*-test. The compounds obtained as a result of Student’s *t*-test were used to create a heatmap. This map was generated using the Euclidean distance measure and the Ward clustering method. Pathway analysis was performed for the metabolites identified in Student’s *t*-test. The hypergeometric test and the KEGG database were selected as enrichment methods.

## 3. Results

**Figure 3:**
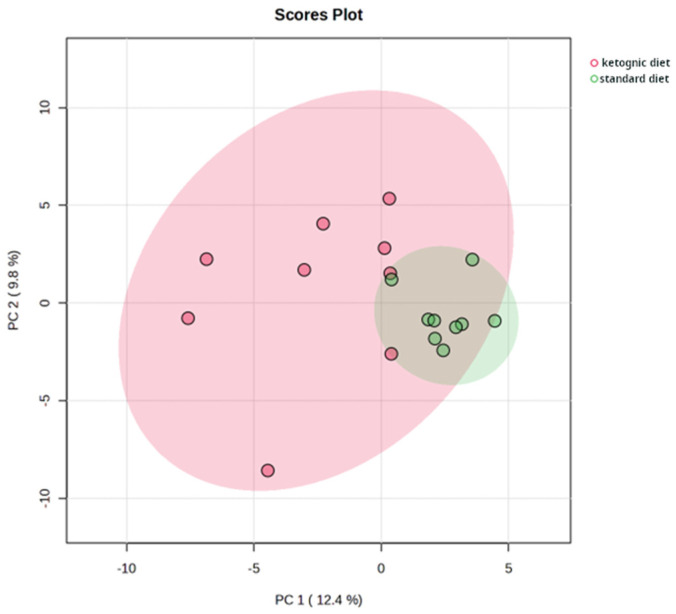
PCA score plot.

**Figure 4:**
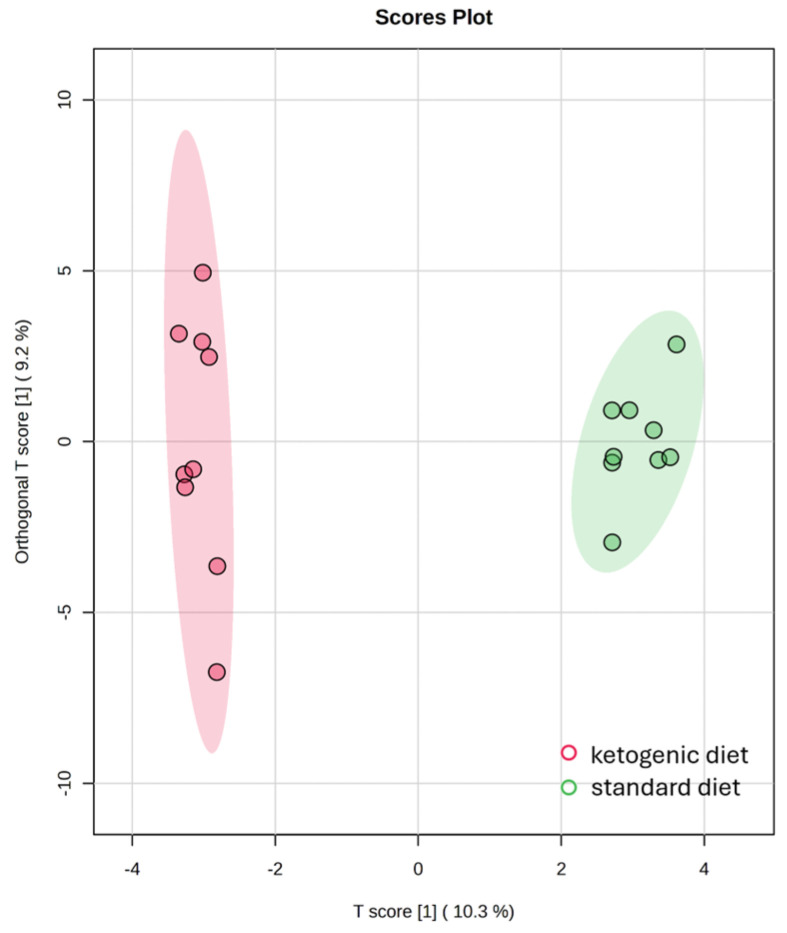
Orthogonal Partial Least Squares Discriminant Analysis (OPLS-DA) score plot showing the separation between participants following the ketogenic diet and the standard diet. The model consisted of one predictive component (p1) and one orthogonal component (o1), explaining 10.6% of the X-variance (R^2^X = 0.105) and 93.7% of the Y-variance (R^2^Y = 0.937), with a predictive ability of Q^2^ = 0.594. Permutation testing (1000 permutations) confirmed the statistical robustness of the model (*p* < 0.001).

After data filtering, 462 annotated GC-MS features were retained for further analysis. Compound annotation was performed by comparing the acquired mass spectra with reference spectra available in the NIST mass spectral library. The initial dataset consisted of 462 annotated GC-MS features assigned putative compound identities based on spectral matching. Subsequently, statistical filtering and multivariate analyses were performed to identify compounds contributing to group discrimination and biological interpretation. PCA analysis (Figure 3) revealed two overlapping clusters, with the more tightly clustered set corresponding to the standard diet. In contrast, OPLS-DA analysis (Figure 4) showed a clear separation between the ketogenic diet and standard diet groups. The model included one predictive component (p1) and one orthogonal component (o1). The predictive component explained 10.5% of the X-variance (R^2^X = 0.105) and 93.7% of the Y-variance (R^2^Y = 0.937), with good predictive ability (Q^2^ = 0.594). Model validation using 1000 permutations confirmed the robustness of the predictive component, as the observed Q^2^ value was significantly higher than expected by chance (*p* < 0.001). To further verify the robustness of the observed group separation, recursive SVM classification was performed using three independent validation approaches. The analysis yielded low classification error rates, reaching 2.8% with 10-fold CV, 0% with LOOCV, and 11.1% with bootstrap validation. These results independently confirmed the discriminatory capacity of the metabolomic profile and supported the stability of the OPLS-DA model (Supplementary Figures S1 and S2). Following application of the VIP method, a panel of 83 compounds with a VIP score > 1, indicating a relatively high contribution to OPLS-DA group discrimination, was generated.

**Figure 5:**
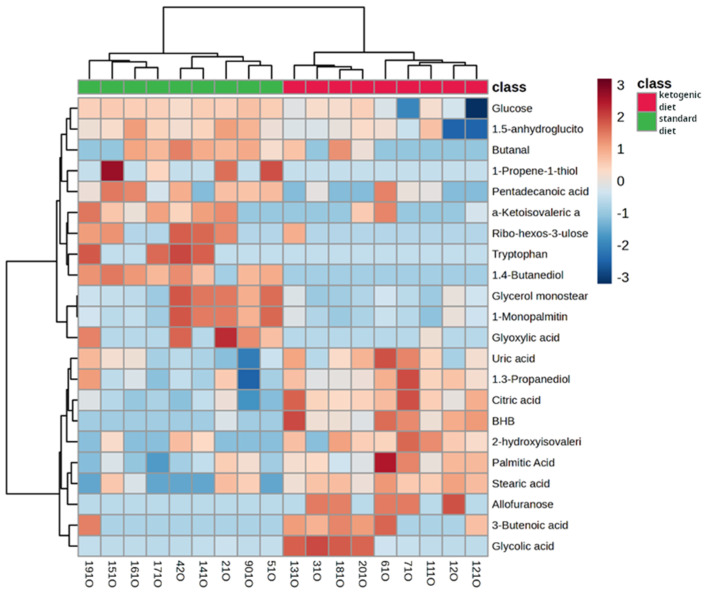
A heatmap accompanied by a hierarchical cluster analysis for 22 metabolites that distinguish the study group from the control group. The compounds were identified using Student’s *t*-test (*p*-value < 0.05).

Student’s *t*-test identified 22 metabolites that differed significantly between groups based on unadjusted *p*-values, whereas only three metabolites remained statistically significant after correction for multiple testing using the Benjamini–Hochberg false discovery rate (FDR) procedure (FDR < 0.05). Given the limited sample size, both FDR-adjusted and unadjusted results were examined; however, only the FDR-significant metabolites should be considered statistically robust findings. The remaining metabolites are presented as exploratory observations and should be interpreted with caution. The distribution and hierarchical clustering of these 22 metabolites are presented in Figure 5. All identified compounds are listed in Table 1.

The presented data show clear differences between the metabolic profiles of patients receiving a ketogenic diet and those receiving a standard diet. β-Hydroxybutyrate (BHB) was the strongest contributor to group discrimination. Differences were also observed for several metabolites related to amino acid metabolism, including tryptophan, 2-hydroxyisovaleric acid, and α-ketoisovaleric acid. However, these metabolites did not remain statistically significant after FDR correction and should therefore be regarded as exploratory findings.

**Figure 6:**
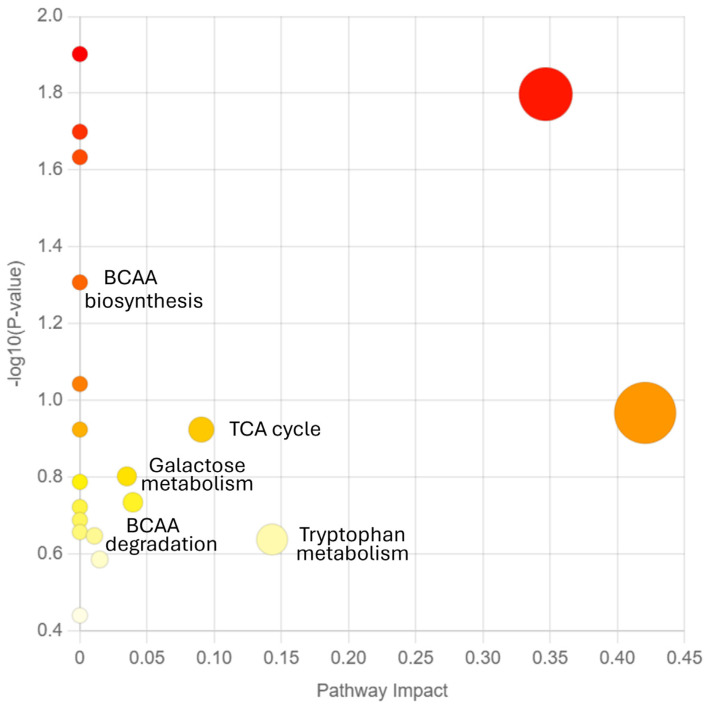
Exploratory pathway analysis based on metabolites showing nominally significant differences in the unadjusted Student’s *t*-test. The color intensity of each circle reflects the *p*-value of the corresponding pathway, with warmer colors indicating lower *p*-values, whereas the circle size represents the pathway impact derived from topology analysis.

Pathway analysis (Figure 6) was performed to facilitate the biological interpretation of the discriminative metabolites. The analysis suggested enrichment of pathways related to energy metabolism and amino acid metabolism, including tryptophan- and branched-chain amino acid (BCAA)-related pathways. Because these analyses were based predominantly on metabolites that did not remain statistically significant after FDR correction, the pathway-level findings should be considered exploratory and require confirmation in larger independent cohorts.

Exploratory correlation analyses between metabolite abundance and seizure frequency were performed in patients with available clinical data (Supplementary Table S1). Although several nominally significant associations were observed, none remained statistically significant after correction for multiple testing.

## 4. Discussion

Metabolomic analysis of plasma samples from children with drug-resistant epilepsy revealed differences between patients on a ketogenic diet and those on a standard diet. These analyses identified a panel of metabolites that discriminated between the two study groups. However, only three metabolites remained statistically significant after correction for multiple testing, whereas the remaining findings should be regarded as exploratory.

**Figure 7:**
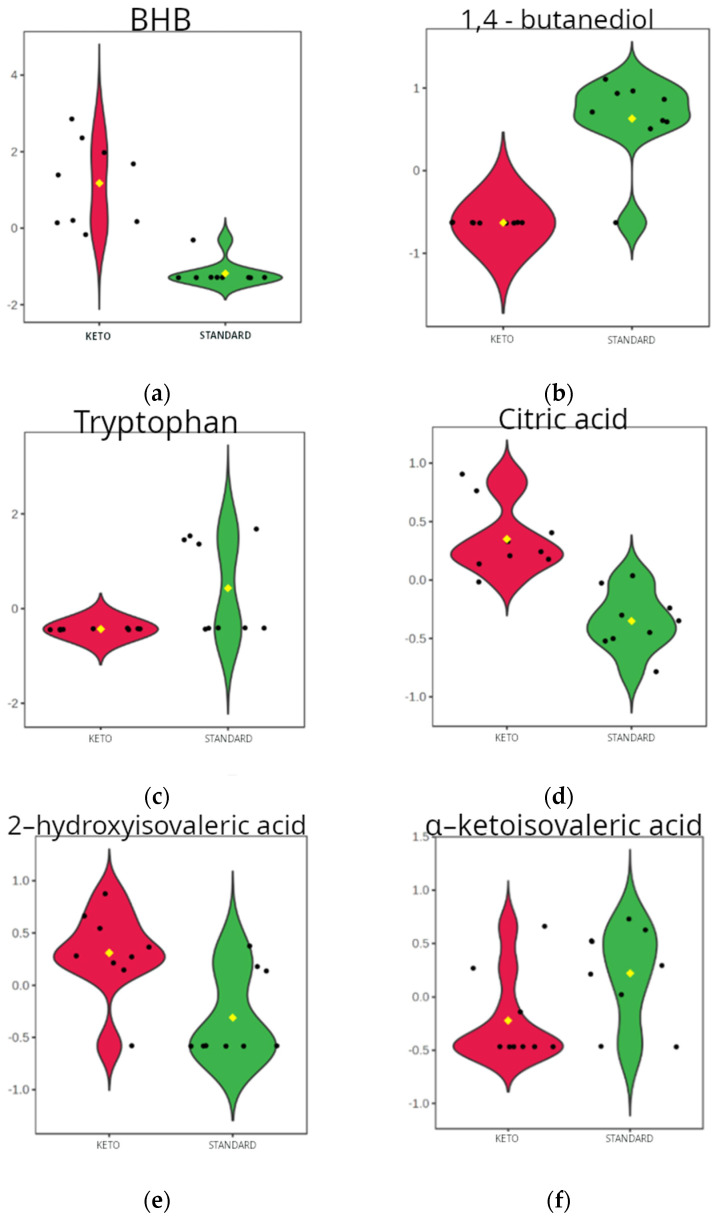
The violin plots of: (**a**) BHB; (**b**) 1,4–butanediol; (**c**) Tryptophan; (**d**) Citric acid; (**e**) 2–hydroxyisovaleric acid; (**f**) α–ketoisovaleric acid. Red violins represent the ketogenic diet group, whereas green violins represent the standard diet group. Black dots indicate individual observations, yellow dots represent the group means, and the width of each violin reflects the density of the data distribution.

The higher abundance of β-hydroxybutyrate (BHB) (Figure 7a) observed in the ketogenic diet group is consistent with the expected metabolic adaptation to ketogenic dietary therapy and reflects increased ketogenesis and fatty acid β-oxidation. These metabolic changes have been widely reported in the literature and have been proposed as contributors to the therapeutic effects of the ketogenic diet [8,22,23]. Qiao et al. (2024) identified BHB as a key metabolite associated with the antiepileptic effects of ketogenic dietary therapy [16]. Experimental and preclinical studies have suggested that ketone bodies may contribute to neuronal membrane stabilization and reduced neuronal excitability, thereby potentially increasing the seizure threshold [8,23]. However, these mechanisms were not directly investigated in the present study and are discussed here only to provide biological context for the observed metabolomic changes. BHB has also been proposed to contribute to neuronal membrane stabilization and to modulate pathways involved in GABA metabolism. Previous experimental studies have suggested that BHB may influence the expression of genes involved in neurotransmitter metabolism, including SIRT4 and GAD1, thereby potentially promoting GABA synthesis [16,17]. Experimental studies have suggested that these mechanisms may increase the GABA/glutamate ratio by promoting GABA synthesis at the expense of glutamate metabolism within the Krebs cycle [16,17]. Yudkoff et al. (2004, 2007, 2008) [24,25,26] proposed several biochemical mechanisms that may explain the observed changes in the GABA/glutamate ratio. They suggested that this phenomenon may result, among other things, from the utilisation of acetyl–CoA—produced during the metabolism of ketone bodies—for citrate synthase, and from the fact that oxaloacetate is utilised whilst the activity of asparagine transaminase is reduced. The authors also proposed that increased glutamate availability for GAD-mediated GABA synthesis, together with reduced transamination to aspartate, may contribute to these metabolic adaptations [24,25,26]. Because GABA and glutamate concentrations were not measured in the present study, these mechanisms cannot be directly evaluated in our cohort and are discussed only as potential biological explanations for the observed metabolomic profile.

The results showed the presence of 1,4–butanediol (1,4–BD) (Figure 7b) exclusively in the control group. Following ingestion, this compound is rapidly converted into γ–hydroxybutyric acid (GHB), a neuroactive molecule that exerts potent effects within the central nervous system [27]. The biotransformation of 1,4-BD occurs through a two-step pathway involving alcohol dehydrogenase (ADH) and aldehyde dehydrogenase (ALDH), resulting in the sequential formation of γ-hydroxybutyraldehyde and subsequently GHB [27,28]. The potential relationship between 1,4-BD and drug-resistant epilepsy is primarily linked to the pharmacological properties of its active metabolite. GHB interacts with both specific GHB receptors and GABA(B) receptors, modulating neuronal excitability as well as GABAergic and glutamatergic neurotransmission. These effects are concentration-dependent. At lower concentrations, GHB predominantly exhibits inhibitory effects, whereas higher concentrations may lead to paradoxical synchronization of neuronal activity and the generation of pathological cortical discharges [29].

The relevance of GHB in epilepsy research has been demonstrated in numerous animal models. Administration of GHB or its precursors, including 1,4-BD, is commonly used to induce absence-like seizures resembling human absence epilepsy. These models are characterized by synchronized spike-and-wave discharges involving corticothalamic circuits, highlighting the influence of GHB on mechanisms regulating neuronal network excitability and rhythmic activity [29].

Clinical observations also suggest a potential association between disturbances in GHB metabolism and seizure occurrence. Cases of generalized tonic–clonic seizures have been reported following acute 1,4-BD intoxication as well as during withdrawal after chronic exposure [28]. High concentrations of GHB may lead to profound central nervous system depression, respiratory impairment, altered consciousness, and secondary electrophysiological disturbances that may promote seizure activity.

However, it should be emphasized that the detection of 1,4-BD exclusively in the control group does not provide direct evidence for either a proconvulsant or protective effect in drug-resistant epilepsy. Given the low abundance of this metabolite and its rapid conversion to GHB, this observation may instead reflect differences in environmental exposure, endogenous metabolism, or the activity of enzymatic pathways involved in GABA and GHB metabolism. Nevertheless, the identification of 1,4-BD is of interest in the context of GABAergic neurotransmission, which plays a central role in epilepsy pathophysiology and mechanisms of drug resistance. The absence of detectable 1,4-BD in the ketogenic diet group could warrant further investigation in larger cohorts to determine whether ketogenic dietary therapy may influence previously unrecognized metabolic pathways related to GABA and GHB metabolism. The study found higher levels of citric acid in the study group compared to the control group. Avalos et al. (2025) [30] focused their attention on a group of 60 individuals, comprising 32 patients with epilepsy and 28 healthy individuals. Using NMR to analyse serum, they demonstrated that patients had lower concentrations of citric acid compared to the healthy group. They also identified a probable cause for these changes. They concluded that these changes in concentration may indicate a disturbance in cellular respiration processes, caused by increased oxidative stress and inflammation within the body [30,31,32]. Wang et al. (2016) [33], also comparing the profiles of patients with epileptic seizures (27 individuals) with those of healthy individuals (23 individuals), demonstrated reduced serum citrate levels (*p* = 0.029). Non-targeted GC-MS analysis, in addition to lower citrate concentrations, revealed, among other things, reduced levels of palmitic acid (*p* < 0.001) and linoleic acid (*p* < 0.001), alongside higher concentrations of lactate (*p* < 0.001), butanoic acid (*p* = 0.005), proline (*p* = 0.004) and glutamate (*p* = 0.005) [33]. Guo et al. (2023) [34] conducted a study on a group of 90 patients. The study group comprised 53 individuals who had responded to valproic acid monotherapy. In contrast, the control group consisted of 37 individuals who had not responded to valproic acid polytherapy. Non-targeted metabolomic and lipidomic analyses of plasma revealed that the citric acid pathway is associated with epileptic seizures. When comparing the two groups, higher concentrations of citric acid were found in those who responded positively to the treatment [34].

Citric acid has so far been associated with drug-resistant epilepsy through its use as a supplement to the ketogenic diet. Most patients on a ketogenic diet receive routine potassium citrate supplementation [35,36,37]. This supplementation is an essential part of treatment to prevent kidney stones, which occur in approximately 3–6% of patients on a KD due to urinary acidosis and hypercalciuria [35,37]. Potassium citrate alkalises the urine and dissolves calcium, drastically reducing the risk of renal complications. Additionally, this compound prevents metabolic acidosis during high-fat diet therapy [36]. By promoting the oxidation of fatty acids, the ketogenic diet leads to the generation of large amounts of acetyl-CoA, which gives it appropriate anaplerotic properties [38]. Supporting the citric acid cycle by providing alternative fuel sources may increase the availability of its intermediates, including citric acid. Experimental studies have suggested that such metabolic adaptations may contribute to neuronal membrane stabilization and an increased seizure threshold [11,38,39]. The higher citric acid level observed in the ketogenic diet group is the opposite of the typical metabolic profile of epilepsy itself, in which a deficiency of this compound predominates [30,32,33,34]. This observation may reflect the therapeutic intervention itself, either through routine potassium citrate supplementation used to prevent nephrolithiasis or through metabolic adaptations associated with ketogenic dietary therapy. However, because potassium citrate supplementation was not evaluated separately in the present study, its contribution to the observed citric acid levels cannot be determined.

The study also identified differences in metabolites related to amino acid metabolism (Figure 6). The presence of tryptophan, observed only in the control group, may indicate differences in amino acid metabolism between groups; however, this finding should be interpreted cautiously, as it did not remain statistically significant after correction for multiple testing. Previous studies have suggested that tryptophan metabolism may be associated with neurological and metabolic alterations observed during epilepsy and ketogenic dietary therapy, particularly through pathways related to serotonin, kynurenine metabolism, and the gut–brain axis [11,20,30,40]. Alongside tryptophan, metabolites related to valine metabolism (Figure 7e,f), one of the branched-chain amino acids (BCAAs), were also observed. These findings may be consistent with previously reported effects of ketogenic dietary therapy on amino acid metabolism, but they should be regarded as exploratory observations requiring validation in larger independent cohorts.

It should also be noted that tryptophan is an essential amino acid whose circulating concentration may be influenced by both the quantity and composition of dietary protein intake. Therefore, differences in long-term dietary patterns, particularly in the consumption of protein-rich foods, may have contributed to the observed variation in tryptophan levels. Although all blood samples were collected after an overnight fast, which likely reduced the impact of short-term dietary intake, the potential influence of dietary protein composition on tryptophan metabolism cannot be completely excluded and should be considered when interpreting these findings. In recent years, studies have emerged confirming specific changes in plasma amino acid levels during a low-carbohydrate diet or ketogenic dietary interventions. Effinger et al. (2023) [41] conducted a study focusing on a 3-week ketogenic diet in a group of 40 healthy adults. The authors reported a statistically significant increase in valine levels (*p* < 0.0001) and in leucine and isoleucine levels (*p* < 0.0001). Additionally, a decrease in the levels of alanine (*p* < 0.0001), glutamine (*p* = 0.002) and proline (*p* = 0.001) was observed [41]. Similarly, recent metabolomic investigations have reported ketogenic diet-associated changes in circulating amino acid profiles, including alterations in BCAA and tryptophan metabolism, supporting the hypothesis that alterations in amino acid metabolism may represent an important component of metabolic adaptation to ketogenic dietary therapy [8]. More recently, Drabińska-Fois et al. (2026) demonstrated in a randomized KETO-MINOX trial that an energy-restricted ketogenic diet was associated with lower circulating concentrations of alanine, methionine, threonine, and tryptophan, together with higher levels of BCAAs and α-aminobutyric acid compared with a standard diet [42]. Although this study was not conducted in patients with epilepsy, it provides additional evidence that ketogenic dietary interventions can substantially modify systemic amino acid profiles.

Studies in animal models have suggested competition between aromatic amino acids (AAAs) and BCAAs at the blood–brain barrier. Osuch et al. (2022) [43] described competition between BCAAs and AAAs (including tryptophan) for LNAA (Large Neutral Amino Acids) transporters in the blood–brain barrier. In rats, an increase in the BCAA/AAA ratio was observed in plasma, accompanied by a decrease in the hippocampus. The authors suggested that the ketogenic dietary therapy may influence tryptophan availability and kynurenine pathway activity [32]. The issue of the BCAA/AAA ratio was also addressed by Jirapinyo et al. (2004) [44], who, in a study of 20 children with DRE, demonstrated that after 10 days, 19 patients showed a significant increase in the BCAA/AAA ratio (*p* < 0.001). This ratio remained elevated throughout the patients’ stay on KD, suggesting that it may represent a metabolic feature associated with ketogenic dietary therapy [33]. However, in the present study, BCAA- and tryptophan-related findings should not be interpreted as validated biomarkers of treatment efficacy, but rather as exploratory observations requiring confirmation in larger cohorts. Several mechanisms have been proposed to explain changes in the BCAA/AAA ratio, including enhanced gluconeogenesis, the glucose-alanine cycle, BCAA accumulation, and competition for LAT1 transporters [45]. A study by Evangeliou et al. (2009) [46] suggested that BCAA supplementation alongside a ketogenic diet may be associated with seizure reduction in some children with epilepsy [35,36]. Nevertheless, our study did not directly assess BCAA supplementation, amino acid fluxes, transporter activity, or brain amino acid concentrations; therefore, mechanistic conclusions regarding seizure regulation cannot be drawn from the present data [46,47].

Since 2005, the article by Dahlin et al. (2005) [48] has remained a fundamental source of knowledge regarding changes in amino acid concentrations in cerebrospinal fluid during KD therapy. Twenty-six children with DRE underwent ketogenic therapy, and clinical samples were collected before the start of the study and, on average, four months after the dietary change. A key finding was that GABA concentrations in cerebrospinal fluid were significantly higher in patients with a high reduction in seizures (>50% reduction) compared to patients in whom no therapeutic effects were observed. In patients with a very good therapeutic response (>90% reduction in seizures), higher GABA concentrations were present even before the introduction of the KD and persisted throughout therapy. A correlation between GABA levels and age was also demonstrated. In children under 5.5 years of age, a much greater increase in GABA levels was observed, accompanied by a marked reduction in glutamate [48,49]. These findings support the hypothesis that amino acid- and neurotransmitter-related pathways may be involved in the response to ketogenic dietary therapy; however, GABA and other neurotransmitters were not directly measured in the present study.

Most research on KD in drug-resistant epilepsy is based on plasma profiling; however, there are publications addressing amino acid excretion in urine. Akiyama et al. (2023) focused on patients following the MCT–KD and a modified Atkins diet. GC-MS and LC-MS analyses of urine samples revealed elevated concentrations of BCAA catabolites, increased concentrations of structural analogues of GABA and lactic acid, and changes in the tryptophan kynurenine pathway [10]. Effinger et al. (2023) demonstrated reduced urinary excretion of carnitine (*p* = 0.0047), reduced excretion of quinolinic acid (*p* = 0.0478), and increased excretion of kynurenic acid (*p* = 0.0269), reflecting a shift toward β-oxidation and ketogenesis accompanied by modification of amino acid metabolism [41]. Recent metabolomic studies in pediatric epilepsy further support the relevance of tryptophan metabolism and the gut–brain axis, although their findings also highlight the complexity and heterogeneity of these pathways. Therefore, the amino acid-related changes observed in the present study should be interpreted as exploratory observations that are consistent with previously reported metabolic adaptations to ketogenic dietary therapy but require confirmation in larger, well-controlled cohorts.

Exploratory analyses were also performed to assess potential associations between metabolite abundance and seizure frequency. Nominal correlations were observed for several metabolites related to glutamatergic signalling, glucose-related metabolism, amino acid metabolism, and fatty acid metabolism. However, none of these associations remained statistically significant after correction for multiple testing. Therefore, these findings should be considered exploratory and hypothesis-generating rather than evidence of a direct relationship between metabolomic alterations and seizure burden or treatment response.

## 5. Study Limitations

Several limitations of this study should be acknowledged. First, the study had an observational design and participants were not randomly assigned to dietary treatment. Ketogenic dietary therapy was initiated based on clinical indications and physician decision-making, reflecting routine clinical practice. Consequently, the study groups differed in several baseline characteristics, including sex distribution, age, epilepsy syndrome, seizure characteristics, and antiepileptic medication regimens. These factors may independently influence plasma metabolomic profiles and therefore represent potential confounders. In particular, the unequal sex distribution between groups may have contributed to the observed metabolic differences.

Second, the relatively small sample size limits the statistical power of the study and may affect the generalizability of the findings. Although the OPLS-DA model was validated using cross-validation, permutation testing, and additional recursive SVM analyses, the results should be interpreted with caution and require confirmation in larger independent cohorts.

Third, detailed quantitative dietary intake records, including complete macronutrient distribution, food composition data, and specific protein sources, were not available for all participants. Therefore, the contribution of individual dietary components to the observed metabolomic changes could not be fully assessed. In particular, the potential influence of dietary protein composition on amino acid-related metabolites, including tryptophan, cannot be excluded.

Another limitation is the considerable variability in the duration of ketogenic dietary therapy among participants, which ranged from 2 to 258 weeks. Metabolic adaptations to ketogenic dietary therapy may differ between short-term and long-term treatment and could therefore have contributed to the observed metabolomic profiles. However, due to the limited sample size, subgroup analyses according to treatment duration could not be performed. Future studies with larger cohorts should investigate the temporal dynamics of metabolomic changes during ketogenic dietary therapy.

Finally, only a limited number of metabolites remained statistically significant after correction for multiple testing. Therefore, several observed metabolomic alterations should be considered exploratory and hypothesis-generating until validated in larger prospective studies. This distinction is particularly important because metabolites identified only on the basis of unadjusted *p*-values may reflect biological trends, but they cannot be regarded as statistically robust biomarkers in the present cohort.

Exploratory correlations between metabolite abundance and seizure frequency were performed only in patients with available seizure-frequency data. Although several nominal associations were observed, none remained significant after correction for multiple testing. In addition, the small sample size and heterogeneity of seizure types prevented reliable analyses stratified by seizure type or treatment response.

Future studies involving larger and more clinically homogeneous cohorts, preferably using prospective longitudinal designs with detailed dietary assessment, are needed to validate and extend the present findings.

## 6. Conclusions

The present study suggests that ketogenic dietary therapy is associated with distinct changes in the plasma metabolomic profile of children with drug-resistant epilepsy.

Ketogenic dietary therapy was associated with expected alterations in energy metabolism, particularly increased β-hydroxybutyrate levels and changes in glucose-related metabolites. These findings are consistent with the expected metabolic shift from glucose-based energy metabolism toward ketone body utilization during ketogenic dietary therapy.

Additional differences were observed in amino acid-related metabolites, including those associated with BCAA and tryptophan metabolism. However, these findings should be interpreted cautiously, as most of them did not remain significant after correction for multiple testing and may be influenced by dietary composition, protein intake, medication use, and clinical heterogeneity.

Overall, the results should be considered exploratory and hypothesis-generating. Further studies in larger, more homogeneous cohorts, preferably with longitudinal follow-up and detailed dietary assessment, are needed to validate these observations and clarify their clinical relevance.

## Figures and Tables

**Table 1:** Metabolites showing nominally significant differences between the ketogenic diet and standard diet groups in Student’s *t*-test based on unadjusted *p*-values (*p* < 0.05), together with OPLS-DA VIP scores and FDR-adjusted *p*-values.

↑/↓ ^1^ Metabolite Name	VIP Score	*p*-Value	FDR Adjusted *p*-Value
↓ 1,4-Butanediol	4.457	<0.001	**0.0007**
↑ BHB	8.324	<0.001	**0.0038**
↑ Citric acid	2.447	<0.001	**0.0077**
↑ Stearic acid	3.832	0.003	0.3312
↑ Palmitic acid	2.399	0.005	0.3975
↑ 2-Hydroxyisovaleric acid	2.18	0.006	0.4206
↑ Allofuranose	3.187	0.006	0.4206
↓ 1-Monopalmitin	3.258	0.011	0.4947
↓ Glycerol monostearate	3.192	0.012	0.4947
↓ Glyoxylic acid	1.908	0.02	0.4947
↑ Glycolic acid	3.906	0.02	0.4947
↓ 1,5-Anhydroglucitol	2.58	0.023	0.4947
↓ Tryptophan	3.06	0.023	0.4947
↓ Glucose	5.308	0.024	0.4947
↑ 3-Butenoic acid	1.629	0.024	0.4947
↓ Pentadecanoic acid	1.758	0.025	0.4947
↑ Uric acid	1.782	0.025	0.4947
↑ 1,3-Propanediol	1.407	0.027	0.4947
↓ Ribo–hexos–3–ulose	3.128	0.03	0.4947
↓ 1-Propene-1-thiol	1.195	0.039	0.4947
↓ Butanal	1.694	0.042	0.4947
↓ α-Ketoisovaleric acid	1.576	0.044	0.4947

^1^ ↑—higher level in ketogenic diet group; ↓—lower level in ketogenic diet group compared with the standard diet group. VIP scores indicate the contribution of individual variables to OPLS-DA group discrimination and do not represent statistical significance. FDR-adjusted *p*-values were calculated using the Benjamini–Hochberg procedure; values below 0.05 are shown in bold.

## Data Availability

The original contributions presented in this study are included in the article. Further inquiries can be directed to the corresponding authors.

## Supplementary Materials

The following supporting information can be downloaded at: https://www.mdpi.com/article/10.3390/nu18142347/s1, Figure S1. Evaluation and validation of the OPLS-DA model. (A) OPLS-DA model performance parameters for the predictive component (p1) and orthogonal component (o1), including R^2^X, R^2^Y and Q^2^. The predictive component showed good explanatory and predictive performance (R^2^Y = 0.937, Q^2^ = 0.594). (B) Permutation test of the OPLS-DA model performed using 1000 permutations. The observed Q^2^ value was significantly higher than expected by chance (Q^2^ = 0.655, *p* < 0.001; 0/1000), supporting the predictive robustness of the model; Figure S2. Recursive SVM classification using three validation approaches: (A) 10-fold cross-validation, (B) leave-one-out cross-validation, and (C) bootstrap validation. The analysis showed low classification error rates across validation approaches, supporting the discriminatory capacity of the metabolomic profile; Table S1. Exploratory Spearman correlation analysis between metabolite abundance and seizure frequency in patients with available clinical data (n = 13). Only metabolites with nominal significance (*p* < 0.05) are presented. None of the observed correlations remained statistically significant after Benjamini–Hochberg FDR correction.

## Abbreviations

The following abbreviations are used in this manuscript:

1,4–BD	1,4–butanediol
AAA	Aromatic amino acids
ACN	Acetonitrile
ADH	Alcohol dehydrogenase
ALDH	Aldehyde dehydrogenase
BCAA	Branched-chain amino acids
BHB	β-hydroxybutyrate
BSTFA	N,O-Bis(trimethylsilyl)trifluoroacetamide
CBD	Cannabidiol
CLB	Clobazam
CZP	Clonazepam
DBS	Deep brain stimulation
DRE	Drug-resistant epilepsy
ESM	Ethosuximide
GABA	γ-aminobutyric acid
GAD1	Glutamate decarboxylase 1
GHB	γ-hydroxybutyric acid
IPA	Isopropanol
KD	Ketogenic diet
LAT1	L—type amino acid transporter 1
LEV	Levetiracetam
LNAA	Large Neutral Amino Acids
LTG	Lamotrigine
PRM	Primidone
SIRT4	Sirtuin 4
TCA cycle	Tricarboxylic acid cycle
TPM	Topiramate
VNS	Vagus nerve stimulation
VPA	Valproic acid
