# Supplementary material for: Metabolomic Profiling of Plasma in Children with Drug-Resistant Epilepsy: The Effect of the Ketogenic Diet on Energy and Amino Acid Pathways
Source: Nutrients. 2026 Jul 17;18(14):2347. doi: 10.3390/nu18142347 (PMC13415213; doi:10.3390/nu18142347)
Supplement: Supplementary file 1 [file nutrients-18-02347-s001.zip › nutrients-4355204-supplementary.pdf]

Supplementary Materials

Metabolomic Profiling of Plasma in Children with Drug-Resistant Epilepsy: The Effect of the Ketogenic Diet on Energy and Amino Acid Pathways

Marta Cieślak, Paulina Gątarek, Piotr Safiński, Lukasz Przyslo and Joanna Kałużna-Czaplińska

Evaluation of the OPLS-DA model

Clear discrimination between the ketogenic diet and standard diet groups was achieved using OPLS-DA. Model performance was evaluated using R^2X, R^2Y, and Q^2 parameters, and robustness was assessed by a 1000-permutation test to exclude overfitting and confirm the significance of class separation.

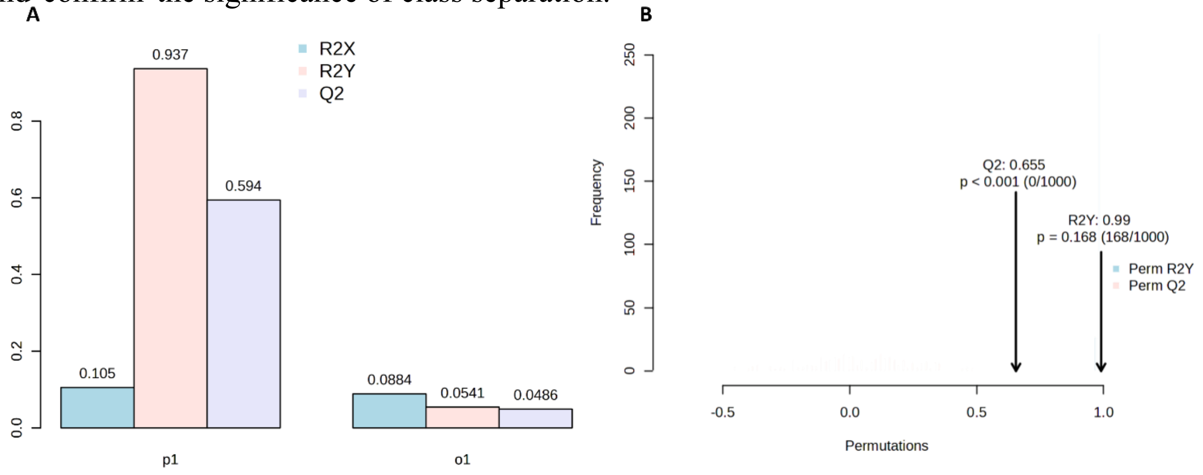

Figure S1. Evaluation and validation of the OPLS-DA model. (A) OPLS-DA model performance parameters for the predictive component (p1) and orthogonal component (o1), including R^2X, R^2Y and Q^2. The predictive component showed good explanatory and predictive performance ($R^2Y = 0.937$, $Q^2 = 0.594$). (B) Permutation test of the OPLS-DA model performed using 1000 permutations. The observed Q^2 value was significantly higher than expected by chance ($Q^2 = 0.655$, $p < 0.001$; 0/1000), supporting the predictive robustness of the model.

Recursive SVM classification

Recursive support vector machine classification was performed as an additional validation approach to assess the robustness of metabolite-based group discrimination. Three validation strategies were applied: 10-fold cross-validation, leave-one-out cross-validation, and bootstrap validation.

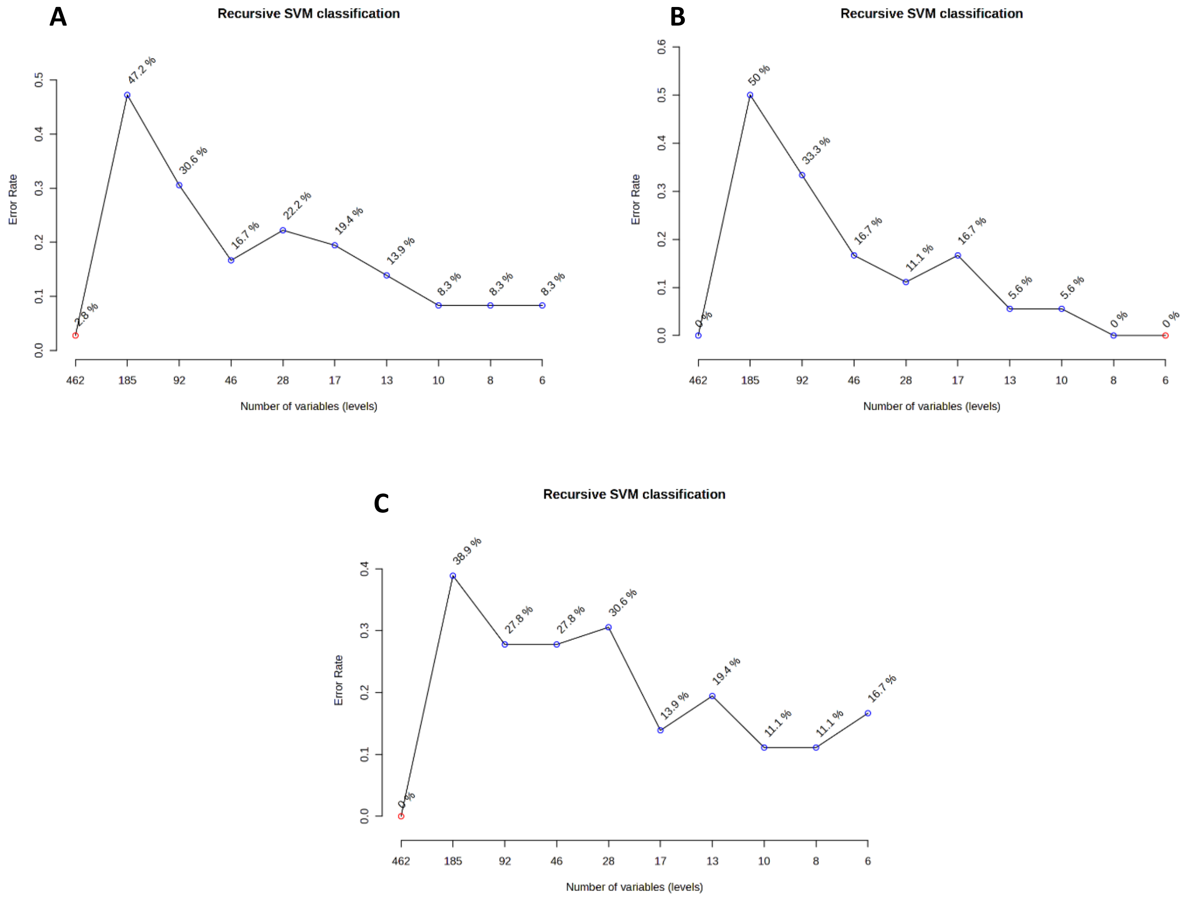

Figure S2. Recursive SVM classification using three validation approaches: (A) 10-fold cross-validation, (B) leave-one-out cross-validation, and (C) bootstrap validation. The analysis showed low classification error rates across validation approaches, supporting the discriminatory capacity of the metabolomic profile.

Exploratory correlation analysis between metabolite abundance and seizure frequency

To explore the potential clinical relevance of the identified metabolites, an exploratory correlation analysis was performed between metabolite abundance and seizure frequency in patients for whom seizure-frequency data were available ($n = 13$). Associations were assessed using Spearman's rank correlation analysis.

Fifteen metabolites showed nominally significant correlations with seizure frequency ($p < 0.05$). The strongest positive correlations were observed for 1-pentamethyldisilyloxycyclopentane, 4-aminophenol, 5-ethyl-dihydro-4,6(1H,5H)-pyrimidinedione, D-ribofuranose, and N-formylglycine ($\rho = 0.776$, $p = 0.0018$). The strongest negative correlation was observed for palmitelaidic acid ($\rho = -0.733$, $p = 0.0044$).

Among the nominally significant metabolites, the most biologically plausible associations with seizure burden were observed for L-glutamic acid (ρ = 0.653, p = 0.0156), L-alanine (ρ = 0.638, p = 0.0190), and several glucose-related features, including D-glucose and β-D-glucopyranose (ρ = 0.634, p = 0.0199). These metabolites are associated with excitatory neurotransmission and energy metabolism, both of which are relevant to epilepsy pathophysiology. In contrast, dodecanoic acid (ρ = −0.638, p = 0.0190) and palmitelaidic acid (ρ = −0.733, p = 0.0044) demonstrated inverse correlations with seizure frequency. However, these metabolites may also reflect dietary exposure and therefore should be interpreted cautiously.

Importantly, none of the observed correlations remained statistically significant after correction for multiple testing using the Benjamini–Hochberg false discovery rate (FDR) procedure. Furthermore, the analysis was limited by the small number of available observations (n = 13), and several metabolites exhibited a high proportion of zero values. Therefore, these findings should be considered exploratory and hypothesis-generating rather than evidence of a direct relationship between metabolomic alterations and seizure burden.

Table S1. Exploratory Spearman correlation analysis between metabolite abundance and seizure frequency in patients with available clinical data (n = 13). Only metabolites with nominal significance ($p < 0.05$) are presented. None of the observed correlations remained statistically significant after Benjamini–Hochberg FDR correction.

Metabolite	Spearman's ρ	p-valu	FDR-adjusted p-valu
1-Pentamethyldisilyloxycyclopentane	0.776	0.0018	0.2258
4-Aminophenol	0.776	0.0018	0.2258
5-Ethyl-dihydro-4,6(1H,5H)pyrimidinedione	0.776	0.0018	0.2258
D-Ribofuranose	0.776	0.0018	0.2258
N-Formylglycine	0.776	0.0018	0.2258
Palmitelaidic acid	-0.733	0.0044	0.3641
Maltose	0.690	0.0091	0.3641
L-Glutamic acid	0.653	0.0156	0.3641
L-Alanine	0.638	0.0190	0.3641
Dodecanoic acid	-0.638	0.0190	0.3641
D-Glucose	0.634	0.0190	0.3641
L-Cystine	0.634	0.0190	0.3641
β-D-Glucopyranose	0.634	0.0190	0.3641

Mercaptoethanol	0.627	0.0218	0.3641
β-D-(+)-Mannopyranose	0.589	0.0343	0.3641

Spearman correlation analysis was performed in patients with available seizure-frequency data ($n = 13$). Although several metabolites showed nominally significant correlations with seizure frequency ($p < 0.05$), none remained statistically significant after Benjamini–Hochberg correction for multiple testing. Therefore, all associations should be considered exploratory and hypothesis-generating.
